# Network pharmacology and molecular docking-based prediction of active compounds and mechanisms of action of Cnidii Fructus in treating atopic dermatitis

**DOI:** 10.1186/s12906-022-03734-7

**Published:** 2022-10-19

**Authors:** Shakeel Ahmad Khan, Ying Wu, Amy Sze-Man Li, Xiu-Qiong Fu, Zhi-Ling Yu

**Affiliations:** Consun Chinese Medicines Research Centre for Renal Diseases, School of Chinese Medicine, Hong Kong Baptist University, Kowloon Tong, Hong Kong, China

**Keywords:** *Cnidium monnieri*, Network pharmacology, Atopic dermatitis, Gene Ontology, KEGG pathway

## Abstract

**Background:**

Atopic dermatitis (AD) is a common inflammatory skin disease that compromises the skin's barrier function and capacity to retain moisture. Cnidii Fructus (CF), the dried fruits of *Cnidium monnieri*, has long been used to treat atopic dermatitis (AD) in China. However, the anti-AD compounds and mechanisms of CF are not fully understood. In this study, we evaluated the active compounds and molecular targets of CF in treating AD.

**Methods:**

The Traditional Chinese Medicine Systems Pharmacology database was used to acquire information regarding the compounds that occur in the herb. Targets of these compounds were predicted using the SwissTargetPrediction website tool. AD-related genes were collected from the GeneCards database. Gene ontology (GO) enrichment analysis and KEGG pathway analysis of proteins that are targeted by active compounds of CF and encoded by AD-related genes were performed using Database for Annotation, Visualization, and Integrated Discovery Bioinformatics Resources. A “compound-target” network was constructed and analyzed using Cytoscape Software. Molecular docking was performed using BIOVIA Discovery Studio Visualizer and AutoDock Vina.

**Results:**

We identified 19 active compounds in CF, 532 potential targets for these compounds, and 1540 genes related to AD. Results of GO enrichment indicated that CF affects biological processes and molecular functions, such as inflammatory response and steroid hormone receptor activity, which may be associated with its anti-AD effects. KEGG pathway analyses showed that PI3K-Akt signaling, calcium signaling, Rap1 signaling, and cAMP signaling pathways are the main pathways involved in the anti-AD effects of CF. Molecular docking analyses revealed that the key active compounds in CF, such as (E)-2,3-bis(2-keto-7-methoxy-chromen-8-yl)acrolein, ar-curcumene, and diosmetin, can bind the main therapeutic targets AKT1, SRC, MAPK3, EGFR, CASP3, and PTGS2.

**Conclusions:**

Results of the present study establish a foundation for further investigation of the anti-AD compounds and mechanisms of CF and provide a basis for developing modern anti-AD agents based on compounds that occur in CF.

## Introduction

Atopic dermatitis (AD)), also known as atopic eczema, is an inflammatory dry skin disease [1, 2]. The frequency and prevalence of AD have steadily grown over the last several decades [1, 3, 4]. AD affects people of all ages but is most often diagnosed during infancy and early childhood. It may persist until maturity and can emerge during adulthood. According to Global Burden of Disease, AD has a prevalence of 15–20% in children and up to 10% in adults, making it the 15^th^ most prevalent nonfatal skin disease. Both males and females are afflicted, and the incidence varies according to race and ethnic group. In the United States, for example, the frequency is much greater among black children (19.3%) than among white children (16.1%) [5, 6]. Around 20% of the population in Hong Kong experiences AD at some point in their lives. Approximately 30% of children in Hong Kong are affected [7–9]. The increased occurrence has been provisionally related to environmental variables such as exposure to air pollution and home hygiene products in high-income and industrialized nations [1]. AD patients often experience itching; their skin can appear dry, cracked and red, and is often thickened. On the cellular level, AD is typically accompanied by keratinocyte apoptosis [10, 11].

AD is thought to be caused by a disruption of the epidermal barrier that allows allergens to penetrate the epidermis and activate dendritic and innate lymphoid cells, which then attract and activate Th2 cells [12]. These activated and dysregulated Th2 cells produce inflammatory cytokines (IL-4, IL-13, and IL-31) in the skin, activating Janus kinase (Jak) pathways. Active Jak pathways activate plasma cells and B lymphocytes, which generate antigen-specific IgE, aggravating the inflammatory response further [13–15].

AD has no definitive cure, and current treatment consists primarily of topical anti-inflammatory agents such as corticosteroids and calcineurin inhibitors. Corticosteroids, powerful anti-inflammatory medicines, have been demonstrated to be beneficial for both acute and chronic AD [11, 16]; however, continuous utilization of topical corticosteroids may result in adverse side effects such as permanent skin thinning and systemic inhibition of adrenal function [11, 17]. Topical calcineurin inhibitors, such as tacrolimus and pimecrolimus, have been shown to ameliorate the symptoms by inhibiting the transcription of important AD-related cytokines (IL-2, IL-4, and IL-5). The US Food and Drug Administration, however, issued an advisory indicating a possible link between calcineurin inhibitors and cancer [18–20]. Traditional Chinese medicine (TCM) has been shown to provide safe, effective alternative immunotherapies for AD [11].

**Fig. 1:**
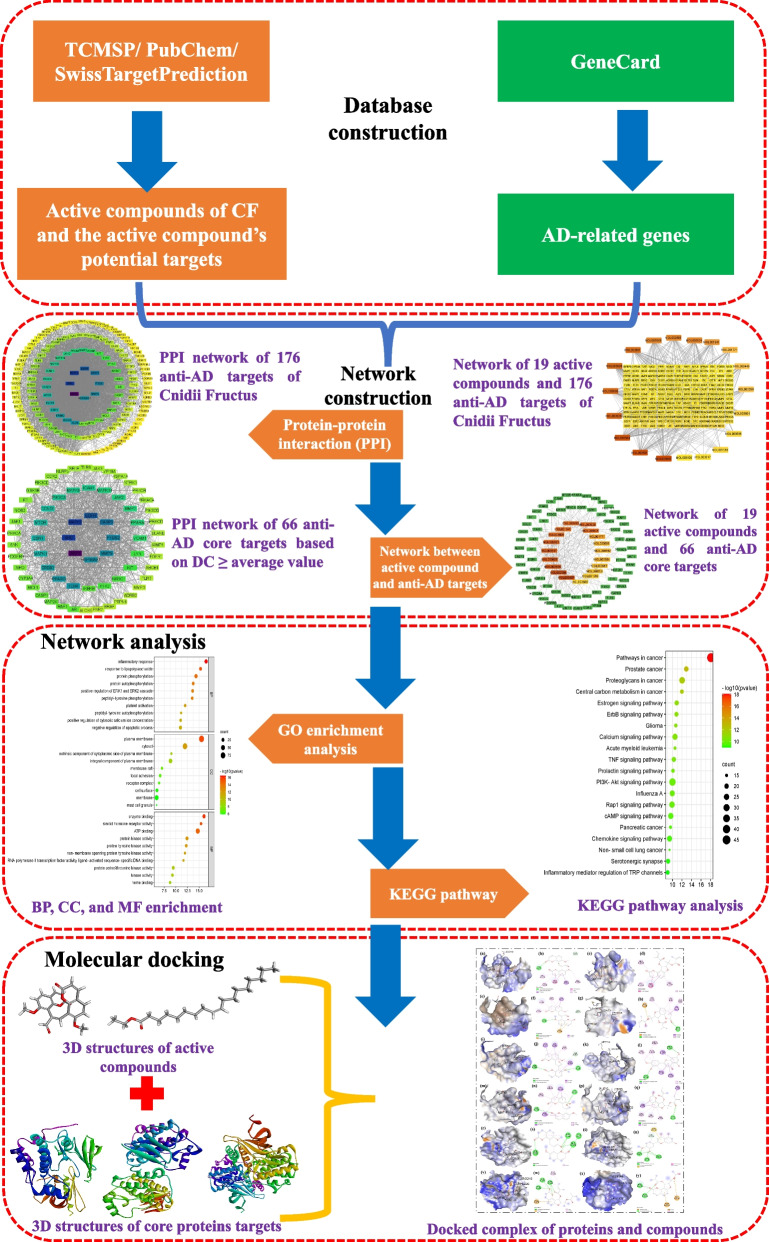
Flowchart of this study

Cnidii Fructus (*She Chuang Zi* in Chinese; common name, Monnier's snow parsley; henceforth referred to here as CF) is the dried fruit of *Cnidium monnieri* (L.) Cuss. The use of CF in China can be traced back to Shennong's Classic of Materia Medica (*Shennong Bencao Jing*) written in the Eastern Han Dynasty (25–220 AD), i.e., close to 2000 years ago. CF is traditionally used for treating female genitals, kidney deficiency, male impotence, and skin-related diseases [21]. Water decoctions and tinctures of CF alone or in combination with other Chinese medicinal herbs are commonly used to treat intractable skin pruritus, superficial fungal diseases, and atopic dermatitis in current TCM clinical practice [22]. More than 400 chemical constituents have been identified in CF. These compounds are mainly categorized as glucides, glycosides, terpenoids, monoterpenoid glucosides, chromones, liposoluble compounds, volatile oils, and coumarins [23, 24]. Pharmacological studies demonstrated that extracts and components of CF have antibacterial, antifungal, anti-osteoporotic, anti-tumor, antiallergic, antipruritic, anti-inflammatory, anti-itching, and anti-dermatitic effects [22, 24]. Total coumarins isolated from CF have recently been reported to have anti-AD effects in a rat model [25]. However, the anti-AD compounds and mechanisms of CF in treating AD have not been fully elucidated. Network pharmacology and molecular docking are now commonly used to identify active pharmaceutical ingredients and understand the overall mechanisms of action of multicomponent herbal drugs [26]. In the present study, we used network pharmacology and molecular docking approaches to predict biologically active phytomolecules, as well as molecular targets and pathways, involved in the anti-AD effects of CF (Fig. 1).

## Materials and methods

### Collection of active compounds of CF

Traditional Chinese Medicine Systems Pharmacology Database and Analysis Platform (TCMSP Version 2.3, https://old.tcmsp-e.com/tcmsp.php, accessed on December 10, 2021) was used to obtain the active phytomolecules of CF [27]. The TCMSP database includes information about the absorption, distribution, metabolism, and excretion (ADME) characteristics of each compound. Oral bioavailability “OB” is a term that refers to "the rate and degree to which an active component or active moiety is absorbed from a therapeutic product that becomes accessible at the targeted site". Drug Likeness “DL” is a qualitative paradigm for drug design that incorporates the ADME qualities of ingredients and established medications. The therapeutic compounds of CF that satisfy the requirements of both OB ≥ 30% and DL ≥ 0.18 [27–30] were collected for subsequent target prediction.

### Target prediction of active compounds of CF

The potential targets of the active compounds of CF (with a probability score > 0) were predicted using the SwissTargetPrediction website tool (http://www.swisstargetprediction.ch/, accessed on December 10, 2021) [31].

### Screening of disease-related genes

The AD-related genes were collected from GeneCards®: The Human Gene Database (https://www.genecards.org/, accessed on December 10, 2021) by searching using the keywords “atopic dermatitis”, “atopic dermatitis inhibiting” and “anti-atopic dermatitis” [32].

### Identification of potential anti-AD targets of CF

Proteins that are compound targets and are encoded by AD-related genes were identified using the Venny 2.1 online software (https://bioinfogp.cnb.csic.es/tools/venny/, accessed on December 11, 2021). The common molecules were considered to be potential anti-AD targets of CF.

### Protein–protein interaction analysis

The protein–protein interaction (PPI) between the potential anti-AD targets of CF was then analyzed by using the STRING database (https://string-db.org/, version 11.5, accessed on December 12, 2021) [33]. The confidence score was set to be > 0.4, and species was limited to “Homo sapiens”. The results of the PPI analysis were further visualized using Cytoscape Software (version 3.9.0, Boston, MA, the USA, accessed on December 14, 2021) [34].

### Compound-target network construction

To analyze the interaction between active compounds and the anti-AD targets of CF, compound-target networks were constructed using Cytoscape Software (version 3.9.0, Boston, MA, USA, accessed on December 14, 2021) [34].

### Enrichment analysis

Gene ontology (GO) functional enrichment analysis and Kyoto Encyclopedia of Genes and Genomes (KEGG) pathway enrichment analysis were carried out using the DAVID database for annotation, visualization, and integrated discovery (Version 6.8; https://david.ncifcrf.gov/, accessed on December 16, 2021) [35–37]. The GO terms were categorized into three types: cellular component (CC), biological process (BP), and molecular function (MF). By uploading the data to the Bioinformatics platform (http://www.bioinformatics.com.cn/), bubble plots of bioprocesses and pathways were drawn. Classical hypergeometric test was used to determine statistical significance. The adjusted *p*-value < 0.05 was utilized as the significance threshold in our investigation after utilizing the Benjamini–Hochberg method to control the false discovery rate (FDR) for multiple hypothesis testing [11].

### Molecular docking

Two-dimensional (2D) structures of active compounds of CF were acquired in Spatial Data File (SDF) format from the NCBI PubChem (https://pubchem.ncbi.nlm.nih.gov/) online database. Their three-dimensional (3D) structures were constructed using BIOVIA Discovery Studio Visualizer 2021 and were saved in PDB format. Protein crystal structures of the potential targets (AKT1, MAPK3, SRC, EGFR, CASP3 and PTGS2) were obtained in PDB format from the Protein Data Bank (https://www.rcsb.org/) [38]. PDB IDs of the six potential targets are 3O96, 6GES, 2BDF, 5Y9T, 1NME and 5IKT, respectievly. BIOVIA Discovery Studio Visualizer 2021 Software was used for the extraction of the ligands and water molecules from the crystal structure complex. It was further employed for designing the grid and the preparation of proteins [39, 40]. The proteins in PDB format were uploaded to AutoDock Vina (version 1.2.0.), and the Kollman charges and Gasteiger partial charges were added to the receptor proteins. The key active compounds in PDB format were then uploaded to AutoDock Vina (version 1.2.0.). Both proteins and key active compounds were converted into pdbqt format using AutoDock Vina (version 1.2.0.). Finally, both proteins and key active compounds in pdbqt format were used for scriptwriting for molecular docking using AutoDock Vina (version 1.2.0.); from this, docked complex results were obtained [41]. The docked complex results were further visualized to estimate the binding ability of the molecules and targets using BIOVIA Discovery Studio Visualizer 2021 software [39]. A binding energy < 0 indicates that a ligand may spontaneously bind to the receptor. It is commonly recognized that the lower the energy score of the ligand and receptor binding configuration, the more probable the binding will occur [41].

## Results

### Active compounds of CF

**Fig. 2:**
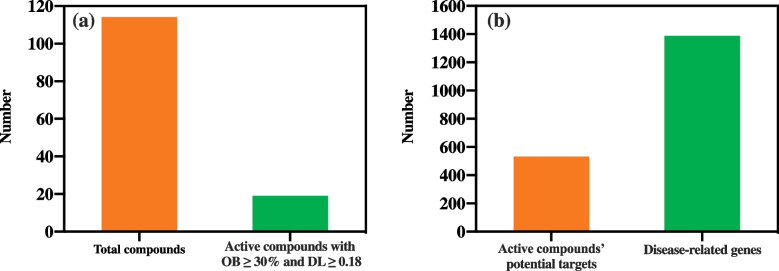
**a** Total numbers of compounds and active compounds present in CF. **b** Total number of active compounds’ potential targets and disease-related genes

A total of 114 compounds of CF were extracted from the TCMSP. Further active compound screening was carried out based on OB ≥ 30% and DL ≥ 0.18; from this, 19 active compounds were obtained (Fig. 2a) as listed in Table 1.

**Table 1:** Active compounds of CF

Serial No	Compound ID	Compound name		OB	DL
		**Common name**	**Standard name**		
1	MOL001510	Campesterol	24-epicampesterol	37.58	0.71
2	MOL001771	Poriferast-5-en-3beta-ol	Poriferast-5-en-3beta-ol	36.91	0.75
3	MOL001941	Imperatorin	Ammidin	34.55	0.22
4	MOL002881	Diosmetin	Diosmetin	31.14	0.27
5	MOL002883	Ethyl oleate	Ethyl oleate (NF)	32.40	0.19
6	MOL000358	Beta-sitosterol	Beta-sitosterol	36.91	0.75
7	MOL003584	Xanthoxyletin	Xanthoxylin N	35.51	0.21
8	MOL003588	Alloimperatorin	Prangenidin	36.31	0.22
9	MOL003591	Ar-curcumene	Ar-curcumene	52.34	0.65
10	MOL003600	Cnidimol B	Cnidimol B	68.66	0.26
11	MOL003604	Cnidimol F	Cnidimol F	54.43	0.28
12	MOL003605	Cnidimonal	(E)-2,3-bis(2-keto-7-methoxy-chromen-8-yl)acrolein	56.38	0.71
13	MOL003606	Cniforin A	Cniforin A	55.89	0.47
14	MOL003607	Cniforin B	Cniforin B	36.70	0.60
15	MOL003608	Columbianetin acetate	*O*-acetylcolumbianetin	60.04	0.26
16	MOL003617	Isogosferol	Isogosferol	30.07	0.25
17	MOL003624	*O*-isovalerylcolum bianetin	*O*-isovalerylcolum bianetin	64.03	0.36
18	MOL003626	Ostruthin	Ostruthin	30.65	0.23
19	MOL000449	Stigmasterol	Stigmasterol	43.83	0.76

*OB* Oral bioavailability, *DL* Drug-likeness

### Targets of CF’s active compounds

To obtain direct targets of the 19 active compounds of CF, ligand-based target prediction was performed using the SwissTargetPredictaion website tool. A total of 1387 potential targets (probability score > 0) were obtained. After eliminating redundancy, 532 predicted potential targets were selected for further analysis (Fig. 2b).

### Disease-related genes

A total of 1540 AD-related genes were obtained by searching in the GeneCards database using the keywords “atopic dermatitis”, “atopic dermatitis inhibiting”, and “anti-atopic dermatitis” (Fig. 2b).

### Intersection of AD-related gene products and compound targets in Venn diagram

**Fig. 3:**
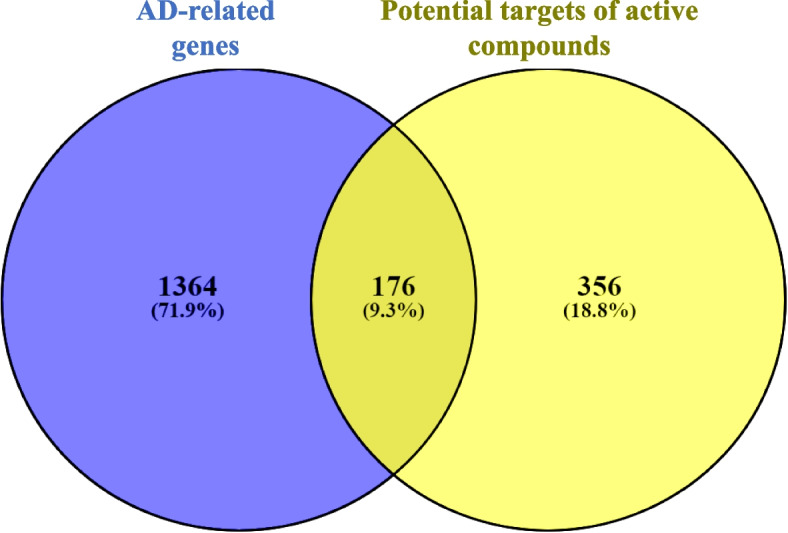
Venn diagram depicting the intersection of AD-related gene products and proteins targeted by CF’s active compounds

Intersection of AD-related gene products and compound targets were identified using the Venny 2.1.0 online system. The results are presented in Fig. 3. A total of 176 compound targets that map with disease-gene products were considered to be potential anti-AD targets of CF.

### Analysis of protein–protein interaction (PPI)

**Fig. 4:**
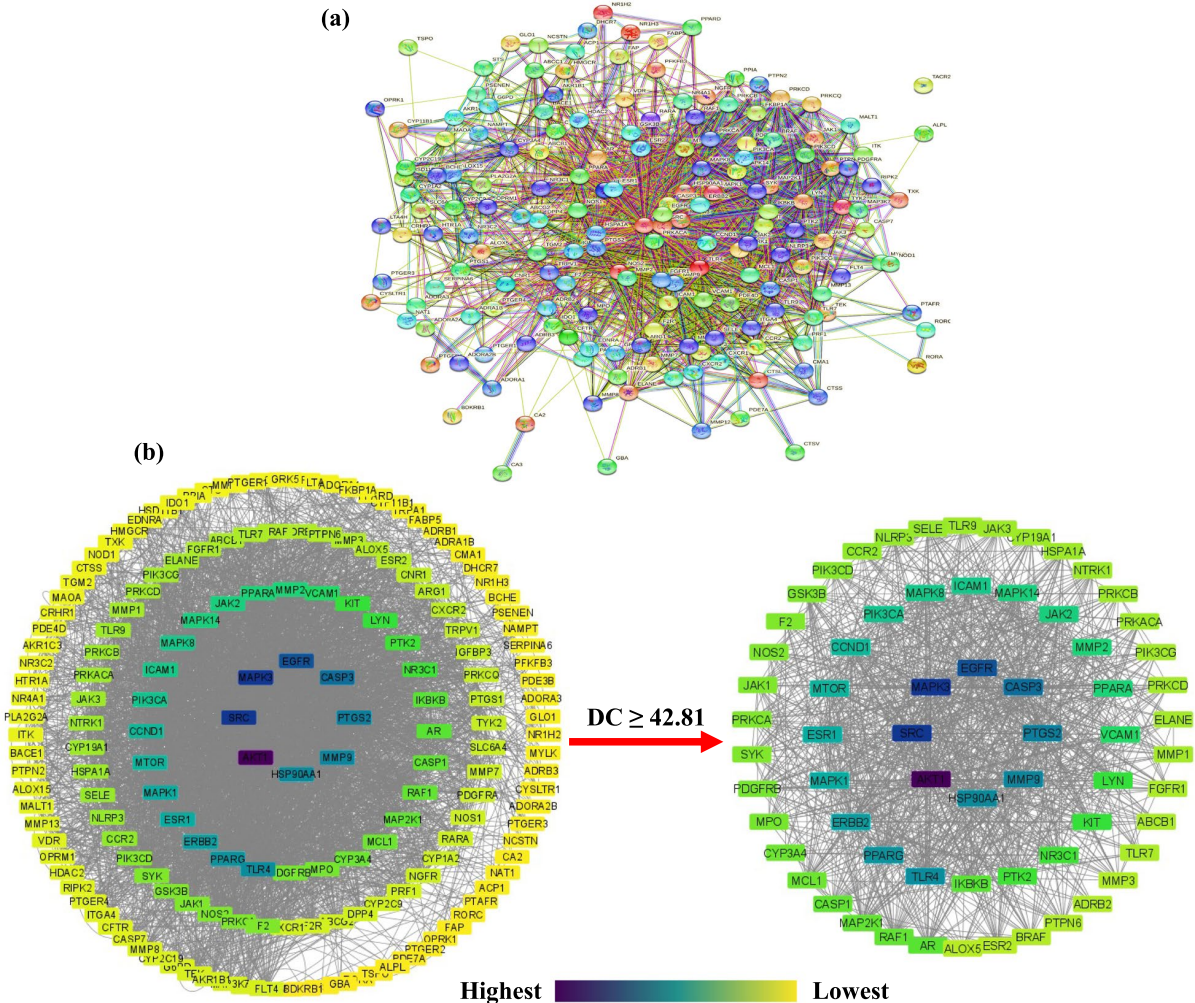
**a** PPI interaction network of 176 potential anti-AD targets of CF constructed using STRING Database 11.5. **b** PPI interaction network of the 176 potential anti-AD targets of CF and the 66 anti-AD core targets constructed using Cytoscape Software 3.9.0. DC denotes degree centrality. Node color represents degree of interaction from yellow (lowest) to purple (highest)

The PPI network of the 176 potential anti-AD targets of CF was constructed by importing them into STRING Database 11.5 (Fig. 4a). The network contained 176 nodes and 1873 edges. Average node degree, average local clustering coefficient, and average PPI enrichment p-values were 21.3, 0.485, < 0.00001, respectively.

**Fig. 5:**
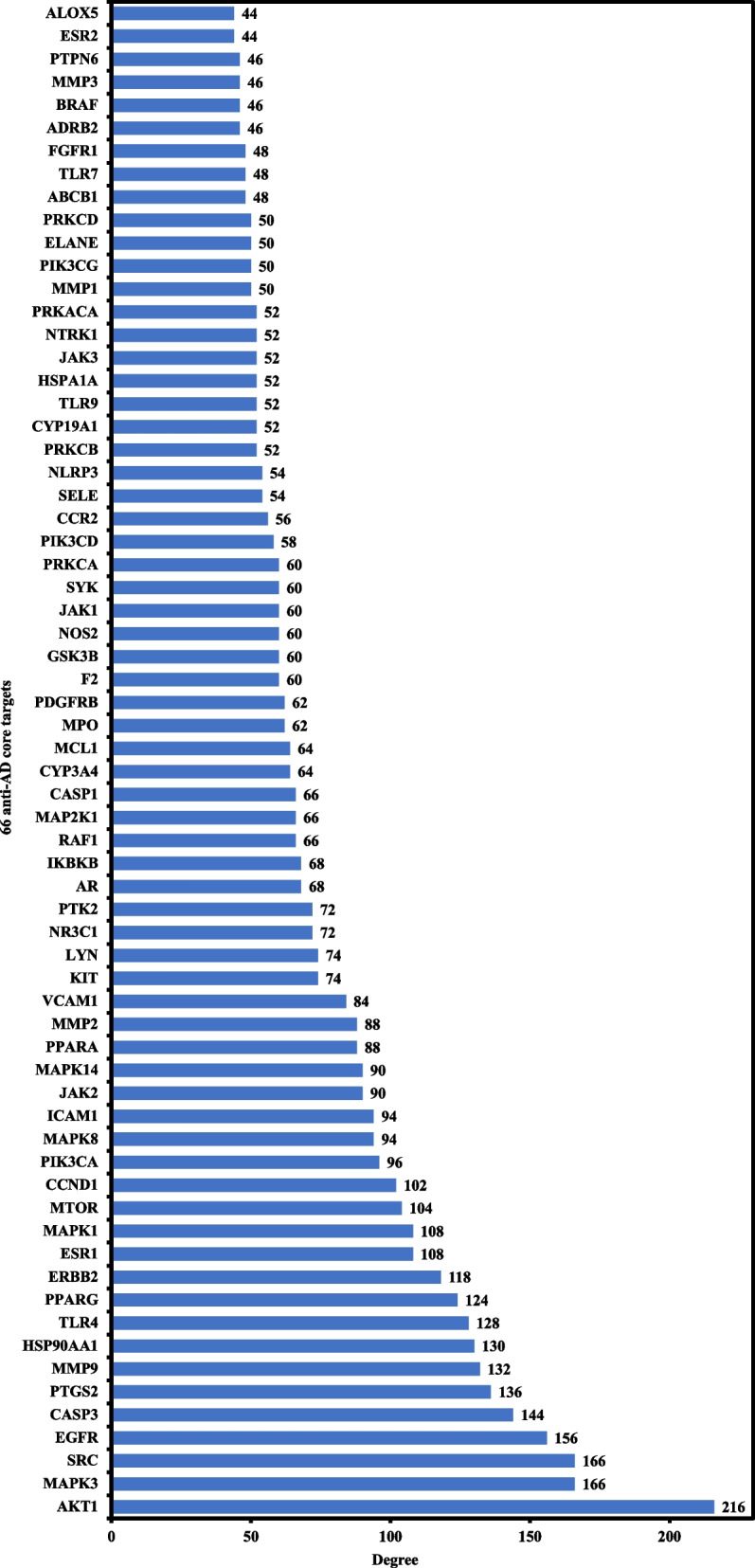
Bar graph of CF’s anti-AD core targets with DC ≥ 42.81

The PPI analysis results were further exported as a simple textual data format (.tsv) file and then imported to Cytoscape software 3.9.0 to obtain the PPI network. The results demonstrated that the network involved 175 nodes and 3748 edges, in which the average shortest path length value between all node pairs in the network was 2.148 with a network radius value of 3. Figure 4b depicts the network with the nodes as colored circles and the color of each node representing its degree, from yellow (lowest) to purple (highest). The 66 nodes that satisfied the criteria of degree centrality (DC) ≥ average value of (42.81) were further extracted and considered as anti-AD core targets (Fig. 4b). Ranked by DC, the 66 anti-AD core targets are presented in the form of a bar graph in Fig. 5. The top 6 anti-AD core targets, namely AKT1, MAPK3, SRC, EGFR, CASP3, and PTGS2, were selected for molecular docking that will be described in Network of active compounds and anti-AD targets of CF section.

### Network of active compounds and anti-AD targets of CF

**Fig. 6:**
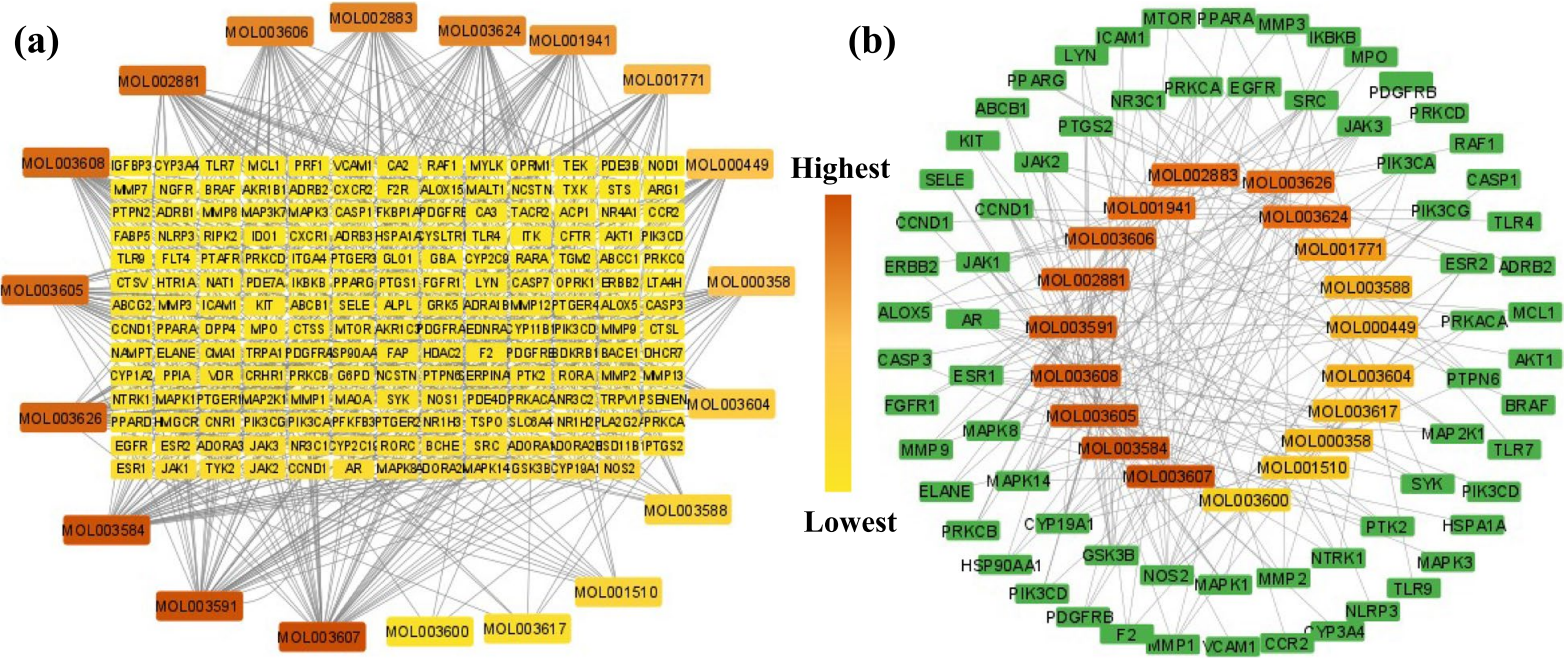
**a** Network of 19 active compounds and the anti-AD targets of CF. **b** Hub network of 66 anti-AD core targets and the 19 active compounds. Node colour represents degree (number of connections), from lowest (yellow) to highest (red orange)

A network displaying the 19 active compounds (determined as described in Active compounds of CF section) acting on the 176 anti-AD targets of CF (determined as described in Analysis of protein-protein interaction (PPI) section) was constructed using Cytoscape Software 3.9.0 (Fig. 6a). Results showed that the network has 201 nodes and 524 edges. Each edge denotes the connection of an active compound to an anti-AD target of CF. The color of each node of compounds shows the degree from lowest (yellow) to highest (red orange). The node degree represents the number of edges connected to this node in the network [42].

**Fig. 7:**
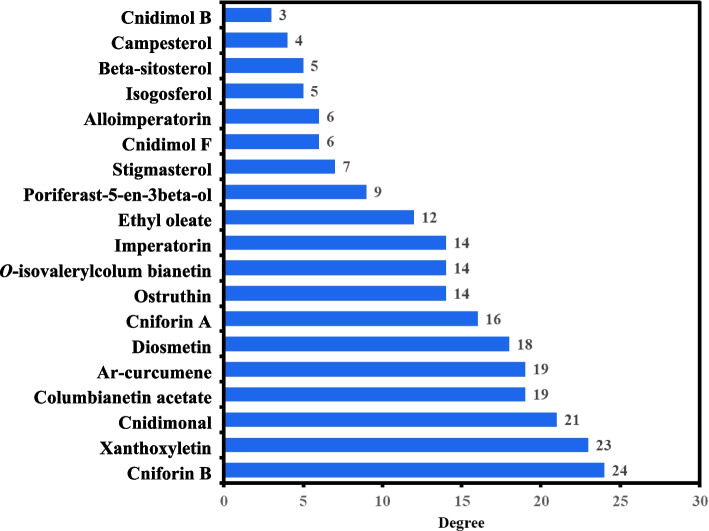
Bar graph of degree values of CF’s active compounds

One more hub network was constructed between the 66 anti-AD core targets (Analysis of protein-protein interaction (PPI) section) and the 19 active compounds, as shown in Fig. 6b. The 66 anti-AD core targets were acted on by all the 19 active compounds. The 19 active compounds with respect to their degrees in the hub network are presented in the form of a bar graph in Fig. 7. Ranked by DC ≥ average value of 12.578, the top 10 active compounds (key active compounds; each has more than 13 targets) are cniforin B, xanthoxyletin, cnidimonal, columbianetin acetate, ar-curcumene, diosmetin, cniforin A, ostruthin, *O*-isovalerylcolum bianetin, and imperatorin (Fig. 7). The network shows that a single compound can interact with multiple targets, and that multiple compounds can interact with a single target. These findings confirm the complexity of the interactions between multiple targets and multiple active compounds in CF.

### GO enrichment analysis

**Fig. 8:**
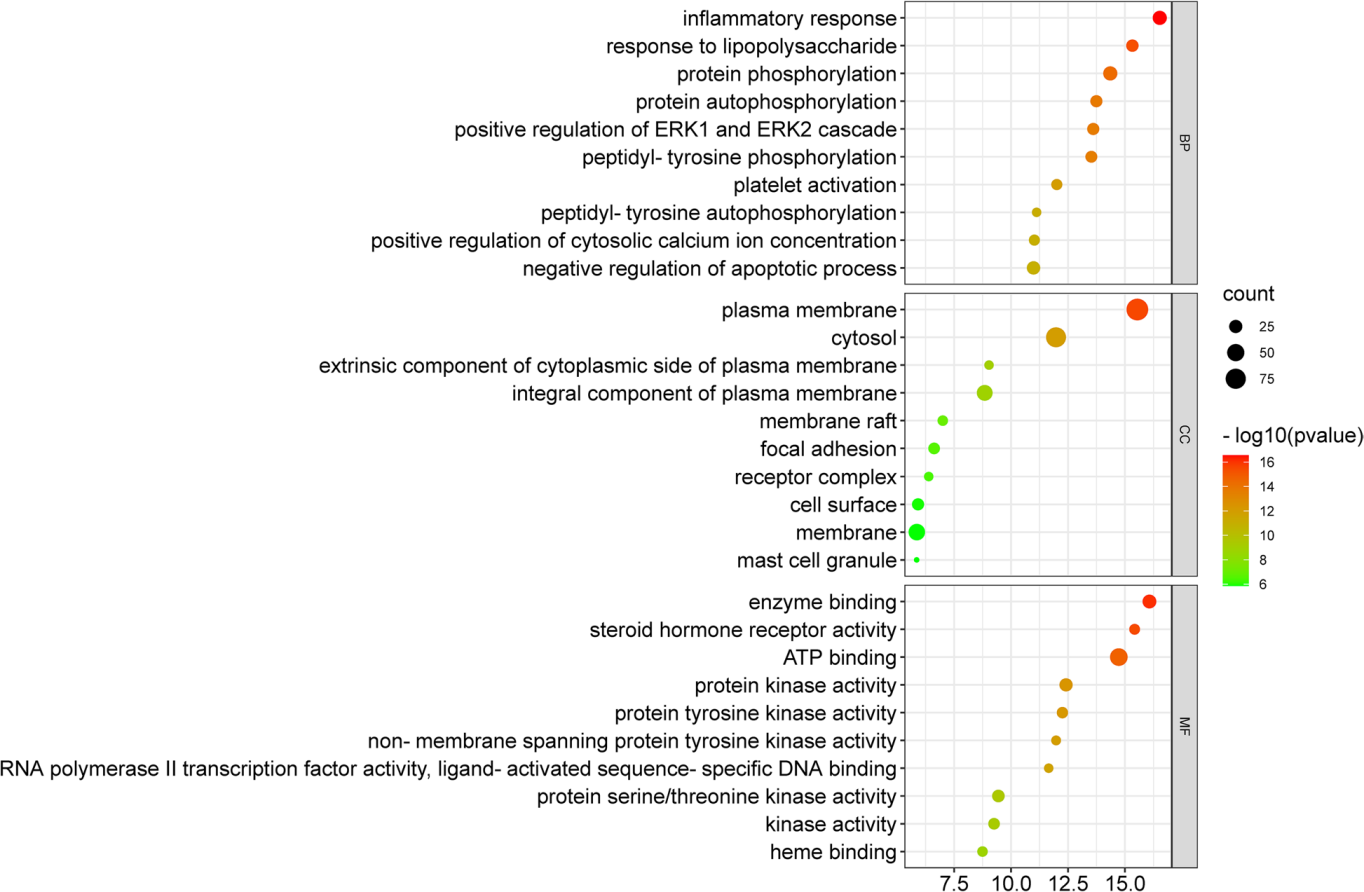
GO enrichment analysis for the 176 anti-AD targets of CF. X-axis presents the number of targets; Y-axis exhibits biological process (BP), cellular components (CC), and molecular function (MF)

GO enrichment analysis was carried out to analyze the 176 anti-AD targets of CF. The respective top 10 enriched terms of BP, CC, and MF of the 176 targets are presented in Fig. 8. The results of GO enrichment analysis demonstrated that anti-AD targets of CF are involved in multiple biological processes, such as inflammatory response, response to lipopolysaccharides, protein phosphorylation, positive regulation of ERK1 and ERK2 cascades, and negative regulation of the apoptotic process. In the cellular component category, anti-AD targets of CF are mainly plasma membrane proteins, cytosol proteins and integral components of the plasma membrane. GO enrichment analysis results showed that the enriched molecular function ontologies are dominated by ATP binding, enzyme binding, proteins kinase activity, and protein serine/threonine kinase activity.

### KEGG pathway analysis

**Fig. 9:**
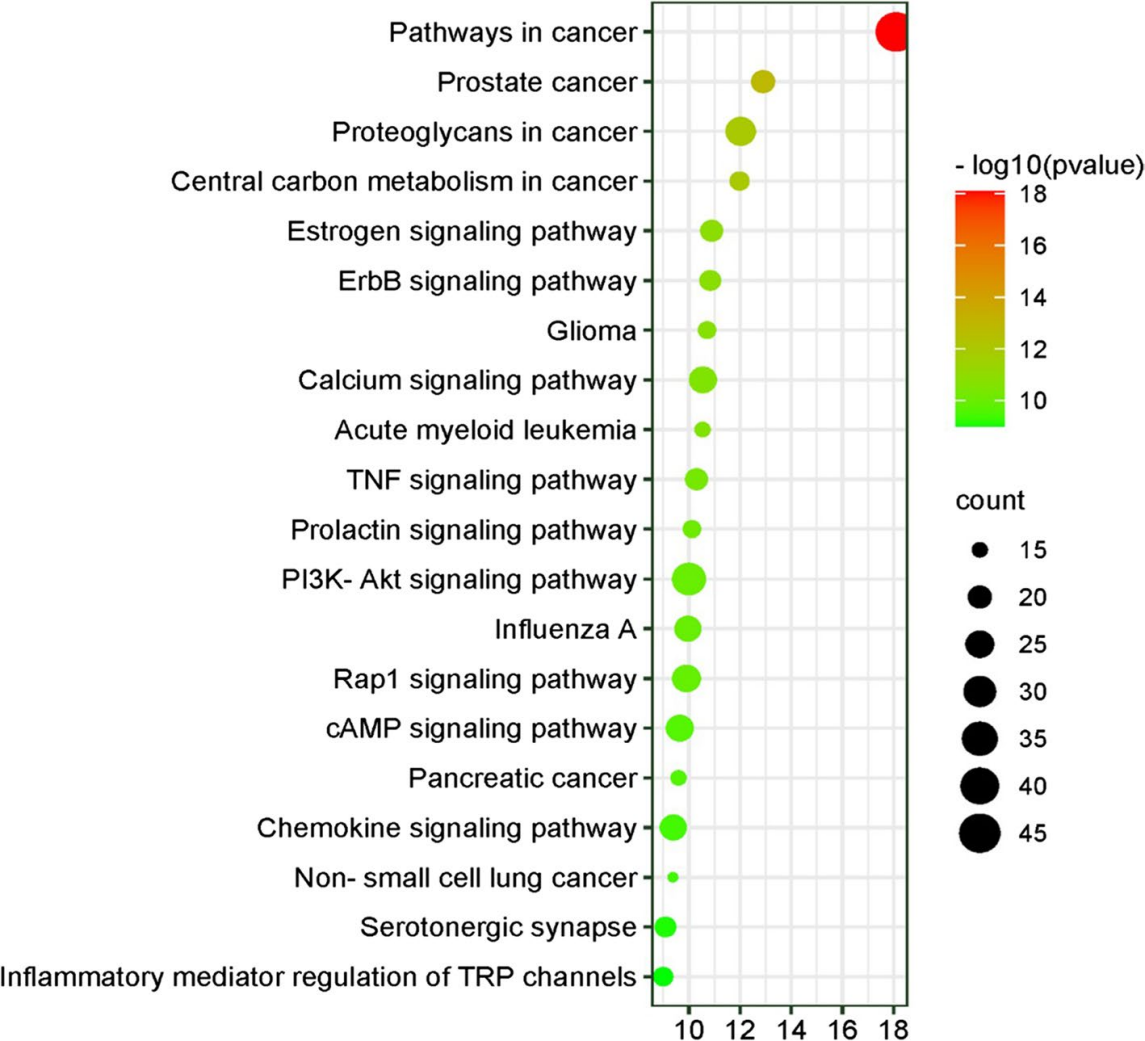
KEGG pathway enrichment analysis for the 176 anti-AD targets of CF. X-axis displays the number of genes; Y-axis displays the various KEGG pathways. Bubble size indicates the number of genes participating in each KEGG pathway

KEGG pathway analysis was carried out to deduce the molecular mechanisms by which CF treats AD. A total of 96 pathways, each with a *p*-value < 0.05, were identified after uploading 176 anti-AD targets of CF to the DAVID platform. The top 20 significant KEGG pathways are presented in Fig. 9. Findings of the KEGG pathway enrichment revealed that anti-AD targets of CF appear to be mainly involved in PI3K-Akt signaling pathway, calcium signaling pathway, Rap1 signaling pathway, cAMP signaling pathway, chemokine signaling pathway, TNF signaling pathway, and inflammatory mediator regulation of TRP channels. These pathways appear likely to play roles in the molecular mechanisms of CF in treating AD.

### Molecular docking

**Fig. 10:**
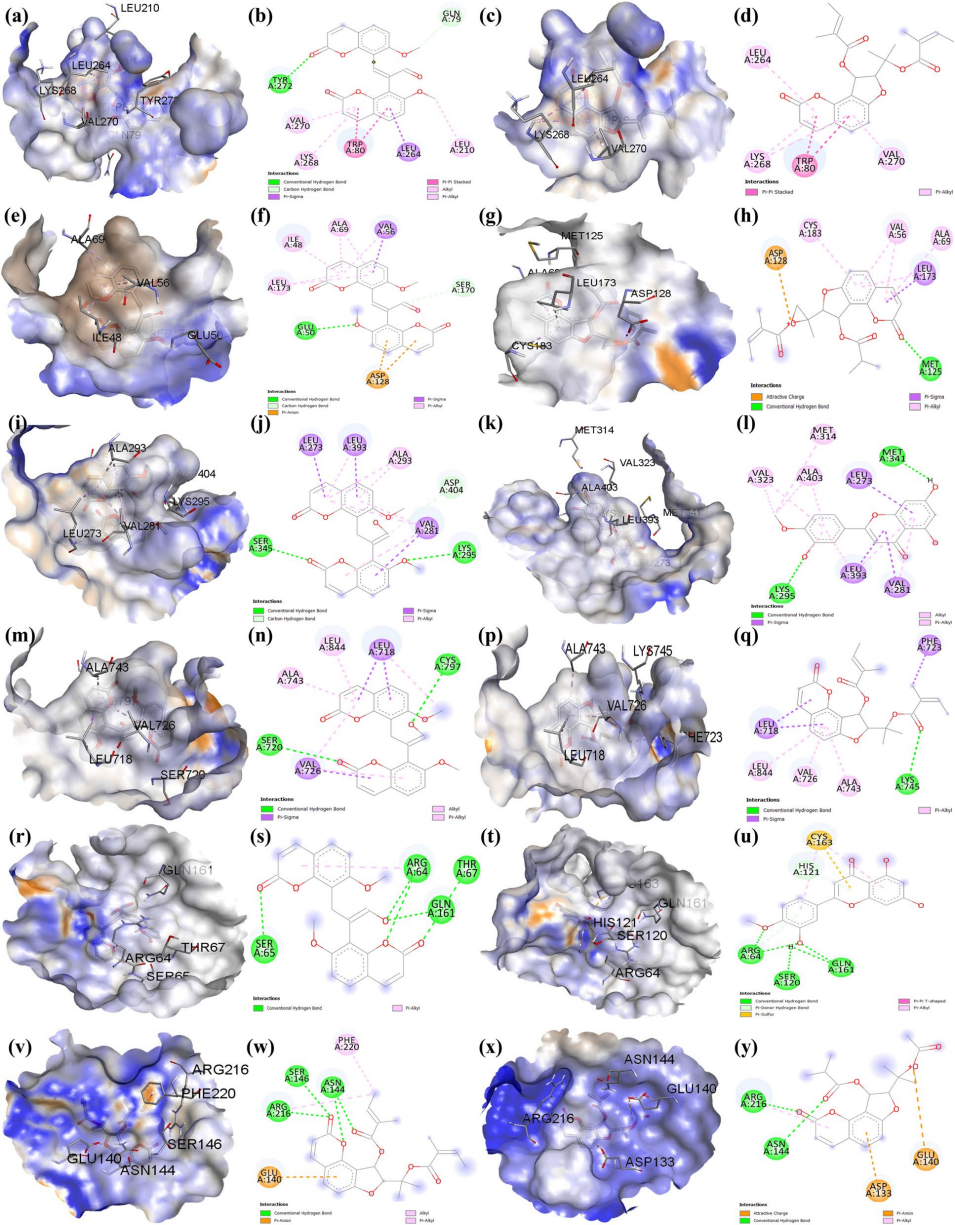
Three D and two D molecular docking results. **a** & **b** Cnidimonal and (**c** & **d**) ar-curcumene bind AKT1. **e** & **f** Cnidimonal and (**g** & **h**) cniforin B bind MAPK3. **i** & **j**)Cnidimonal and (**k** & **l**) diosmetin bind SRC. **m** & **n** Cnidimonal and (**p** & **q**) ar-curcumene bind EGFR. **r** & **s** Cnidimonal and (**t** & **u**) diosmetin bind CASP3. **v** & **w** Ar-curcumene and (**x** & **y**) cniforin A bind PTGS2

Molecular docking of the key active compounds of CF with CF’s anti-AD core targets (AKT1, MAPK3, SRC, EGFR, CASP3 and PTGS2) was conducted. Results are presented in Table 2 and Fig. 10 (a-y).

**Table 2:** Molecular docking of the key active compounds of CF with CF’s anti-AD core targets

Molecule ID	Compound name	Binding energy (kcal/mol)					
		**AKT1**	**MAPK3**	**SRC**	**EGFR**	**CASP3**	**PTGS2**
MOL003608	Columbianetin acetate	-9.7	-7.3	-8.3	-6.9	-5.2	-4.7
MOL002881	Diosmetin	-9.4	-7.8	-8.7	-7.3	-6.2	-4.9
MOL003584	Xanthoxyletin	-9.8	-7.4	-8.3	-6.9	-5.3	-4.4
MOL003591	Ar-curcumene	-10.5	-7.8	-8.4	-8.3	-6.1	-5.1
MOL003626	Ostruthin	-9.5	-7.6	-7.8	-6.7	-5.1	-4.1
MOL003606	Cniforin A	-9.7	-7.6	-8.4	-7.2	-5.2	-5.0
MOL003624	*O*-isovalerylcolum Bianetin	-9.9	-7.2	-8.5	-7.5	-5.4	-4.5
MOL003607	Cniforin B	-9.7	-7.9	-8.6	-7.3	-5.7	-4.9
MOL001941	Imperatorin	-9.0	-7.2	-8.1	-6.4	-6.0	-4.2
MOL003605	Cnidimonal	-10.8	-8.1	-8.7	-8.1	-6.2	-5.0

Binding affinity between a compound and a protein is represented by bingding energy score. The lower the energy, the better the ligand-receptor protein binding is [11]. Docking results demonstrate that each of the key active compounds of CF binds AKT1, MAPK3, SRC, EGFR, CASP3, and PTGS2. These results suggest that the key active compounds of CF directly act on the anti-AD core targets AKT1, SRC, MAPK3, EGFR, CASP3, and PTGS2. The molecular docking findings were consistent with the network pharmacology screening, confirming the trustworthiness of network pharmacology in this study.

## Discussion

The present study explored the active compounds and molecular mechanisms of CF in treating AD. AD is the most common inflammatory skin condition, and multiple signaling pathways potentially contribute to the disease's aetiology simultaneously.

Out of a total of 114 compounds, 19 active compounds with OB ≥ 30% and DL ≥ 0.18 were selected (Table 1) for intensive investigation. Most of the active compounds were found to be multitargeting. The network results demonstrate that ten compounds act on more than 13 anti-AD core targets; these are cniforin B, ar-curcumene, xanthoxyletin, ostruthin, cnidimonal, columbianetin acetate, diosmetin, cniforin A, O-isovalerylcolum bianetin, and imperatorin (Fig. 7). The ten compounds were regarded as key anti-AD compounds of CF. These compounds have satisfactory OB values, thus they are supposed to be orally effective. For treating AD, CF is traditionally used orally and/or topically [21, 22]. Whether the key active compounds of CF can be developed into oral and/or topical anti-AD medications warrants further studies.

The PPI analysis revealed that numerous genes are involved in CF’s effects in treating AD, including AKT1, MAPK3, SRC, EGFR, CASP3, and PTGS2. Reports have demonstrated that inhibiting AKT1 in the skin results in a shift in protease expression, a drop in filaggrin expression, and ultimately, the disintegration of the barrier function of the skin [11, 43]. IL-17 induces the activation of MAPKs that are implicated in the pathophysiology of inflammatory skin disorders [44, 45]. SRC plays a vital role in the activation and proliferation of T cells, leading to severe and chronic AD [46]. Chronic inflammatory skin diseases such as AD have also been associated with the upregulation of EGFR and its ligand expression. Recent research has shown that EGFR-targeted therapy protects against AD by reducing keratinocytes' IL-6 production in response to allergens [47, 48]. CASP3 is regarded as the principal executor and is involved not only in apoptosis pathways but also in inflammation, embryonic and hematopoietic stem cell differentiation. CASP3 plays an important role in keratinocyte apoptosis, which has been implicated as a fundamental cause of spongiosis in AD [49]. COX activity helps to maintain body homeostasis, and inducible COX-2 (PTGS2), in particular, seems to be involved in a number of pathological disorders, including inflammation and pain [50].

GO enrichment analysis demonstrated that compounds in CF may affect a variety of biological processes, including inflammatory response, response to lipopolysaccharides, protein phosphorylation, positive regulation of the ERK1 and ERK2 cascade, and negative regulation of the apoptotic process, all of which may be associated with its anti-AD effects (Fig. 8). Although apoptosis is necessary for cell renewal, excessive chemokines and cytokines produced by skin infection in patients with AD often result in considerable keratinocyte death [43]. Active compounds of CF have been shown to decrease apoptosis in AD, hence promoting keratinocyte regeneration [11, 47].

The KEGG pathway analysis results demonstrated that anti-AD key active compounds of CF act on several signaling pathways, including PI3K-Akt signaling pathway, calcium signaling pathway, Rap1 signaling pathway, cAMP signaling pathway, chemokine signaling pathway, TNF signaling pathway, and inflammatory mediator regulation of TRP channels (Fig. 9). PI3K-Akt signaling pathway dysregulation in the skin leads to a variety of clinical diseases characterized by uncontrolled cell proliferation, such as skin cancer, psoriasis, and AD [51]. Thus, suppressing the PI3K-Akt pathway may be a therapeutic option for AD. The dysregulation of the calcium signaling pathway has been reported to occur when AD is induced, as this pathway controls keratinocyte differentiation and skin barrier formation [52]. Regulating calcium can be another potential strategy in treating AD. TRP channels are regulated by inflammatory mediators and are implicated in the activation-induced release of pro-inflammatory cytokines from keratinocytes; hence, their inhibition may also be advantageous in treating AD [53]. Given that the role of TNF in the etiology of AD is well established [54], targeting TNF is a viable therapeutic strategy for AD. Molecular docking results further confirmed that key active compounds of CF had a regulatory impact on AD-related molecules, such as AKT1, SRC, MAPK3, EGFR, CASP3, and PTGS2.

## Conclusions

The present study explored the compounds and molecular mechanisms active in the therapeutic application of CF to treat AD. In this research, 10 key active compounds of CF and 66 anti-AD core targets were identified. Our study revealed that the underlying mechanisms of anti-AD effects of CF are likely to involve inhibition of the inflammatory response, response to lipopolysaccharides, protein phosphorylation, negative regulation of apoptotic processes, and positive regulation of the ERK1 and ERK2 cascades. Further, we found that four key signaling pathways are likely to be involved: PI3K-Akt signaling pathway, calcium signaling pathway, Rap1 signaling pathway, and cAMP signaling pathway. Our results justify the conclusion that the anti-AD effects of CF might be due to the direct or indirect synergistic effects of multi-target and multi-pathway efforts. Molecular docking results showed that key active compounds of CF can potentially bind to AKT1, SRC, MAPK3, EGFR, CASP3, and PTGS2. Results of the present study establish a foundation for further investigation of the anti-AD compounds and mechanisms of CF and provide a basis for developing modern anti-AD agents based on compounds that occur in CF.

## Data Availability

The datasets used and analysed during the current study available from the corresponding author on reasonable request.
